# Bilateral Renal Vein Thrombosis in Membranous Nephropathy: Hypoalbuminemia Predictive of Venous Thromboembolism in Nephrotic Syndrome

**DOI:** 10.7759/cureus.30032

**Published:** 2022-10-07

**Authors:** Derek Casey, Kaitlyn Romero, Radhika Patel, Taylor Ouellette, Sheela Anasseri, Parham Eftekhari

**Affiliations:** 1 Internal Medicine, Broward Health, Fort Lauderdale, USA; 2 Internal Medicine, Nova Southeastern University Dr. Kiran C. Patel College of Osteopathic Medicine, Fort Lauderdale, USA; 3 General Surgery, Nova Southeastern University Dr. Kiran C. Patel College of Osteopathic Medicine, Fort Lauderdale, USA; 4 Osteopathic Medicine, Nova Southeastern University Dr. Kiran C. Patel College of Osteopathic Medicine, Fort Lauderdale, USA; 5 Nephrology, Broward Health, Fort Lauderdale, USA

**Keywords:** pulmonary emboli, renal vein thrombosis, anticoagulation, membranous nephropathy, hypoalbuminemia, hypercoagulability, venous thromboembolism, nephrotic syndrome

## Abstract

Nephrotic syndrome is a known clinical syndrome in which there is increased permeability in the glomerular basement membrane leading to proteinuria, >3.5g/24h, and hypoalbuminemia. The primary causes of nephrotic syndrome include membranous nephropathy, focal segmental glomerulosclerosis, and minimal change disease. Secondary causes include lupus nephritis, diabetes mellitus, multiple myeloma, amyloidosis, and other systemic conditions. Clinically, nephrotic syndrome presents with edema, hyperlipidemia, and increased risk of thromboembolism, the primary focus of this paper. Nephrotic syndrome is often associated with thromboembolic events, especially in patients with membranous nephropathy. It has been shown that hypoalbuminemia is the most significant independent predictor of venous thromboembolic risk. We present the case of a 32-year-old male who first presented with pleuritic chest pain and was found to have multiple bilateral pulmonary emboli treated with oral anticoagulation. On subsequent visits, prompted by either chest pain or edema, he was found to have increasing pulmonary emboli, as well as downtrending serum albumin levels at each visit. Eventually, bilateral non-occlusive renal vein thrombi were discovered. Lab work indicated membranous nephropathy as the most likely etiology secondary to the patient’s presentation. Serum anti-phospholipase A2 receptor antibody positivity confirmed the diagnosis, and the patient was treated appropriately.

## Introduction

Nephrotic syndrome, defined by the constellation of signs and symptoms including proteinuria >3.5 g/24 hours, hypoalbuminemia, peripheral edema, and hyperlipidemia, is often associated with thromboembolic events (TE) [1]. The symptoms found in patients with nephrotic syndrome are due to the increase in the filtration of intermediate-sized proteins and macromolecules (40-200kDa) into the urine, such as albumin, immunoglobulins, and hormones. Mediators of the clotting cascade are filtered through the urine, such as antithrombin III, protein C, and protein S. The liver then increases the production of factors associated with coagulation to compensate for the loss of platelets, fibrinogen, and factors VIII and X. Patients with nephrotic syndrome are at increased risk of thromboembolic events in both the venous and arterial systems [2].

These TEs may be a preventable cause of morbidity and mortality, particularly in patients with membranous nephropathy (MN), who are at especially high risk. In a study comprised of 898 biopsy-proven MN, 7.2% had at least one venous thromboembolic event (VTE), and hypoalbuminemia, particularly < 2.8 g/dL, was shown to be the most significant independent predictor of VTE [3]. There is a reported incidence of about 15% of deep vein thrombosis (DVT), 10-30% of pulmonary embolism, and 25-37% of renal vein thrombosis. The topic of whether to start prophylactic anticoagulation is controversial, but there is support showing that the risk of thrombotic events may be reduced for patients with nephrotic syndrome, particularly with the primary cause of membranous nephropathy [4]. 

This case report was previously presented at the 2022 Florida Medical Association (FMA) Annual Meeting on August 6, 2022.

## Case presentation

**Figure 1:**
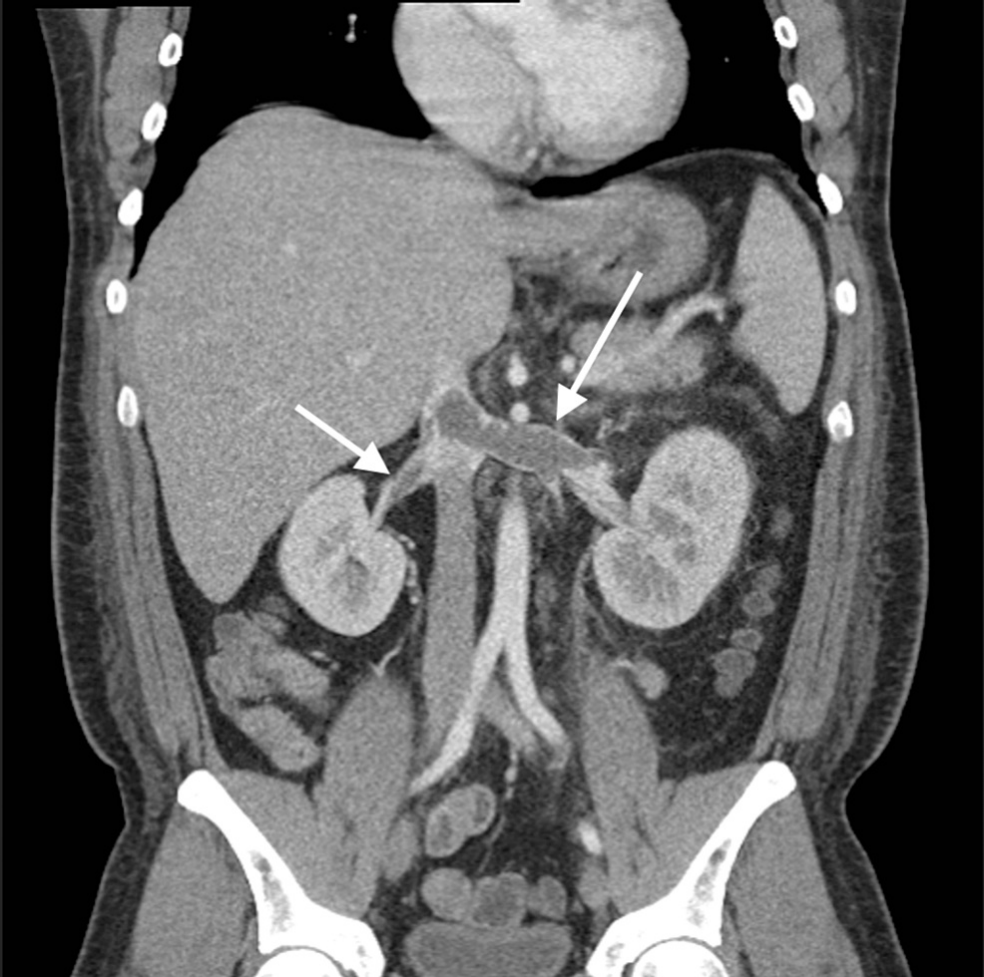
CTPA Demonstrating Bilateral Non-Occlusive Renal Vein Thromboses CTPA: computed tomography pulmonary artery

A healthy 32-year-old man presented to the hospital with left-sided pleuritic chest pain and was subsequently diagnosed with multiple bilateral pulmonary emboli (PE). After evaluation by hematology, and an unremarkable hypercoagulable workup, he was discharged on oral anticoagulation. Labs during this hospitalization included serum albumin of 2.0 g/dL. After completion of oral anticoagulation, repeat CT pulmonary artery (CTPA) six months later showed a minimal amount of retained PE; significantly improved from the considerable amount of emboli seen prior. A few months later he re-presented to the hospital with chest pain, and a third CTPA was performed showing an increasing amount of PE, and an inferior vena cava (IVC) filter was placed. Upon follow-up, signs of nephrotic syndrome were noted by the patient’s primary care physician demonstrated by multiple urinalyses with 300 to >600 mg/dL proteinuria, serum albumin ranging from 1.1-2.1 g/dL, hemoglobin A1c (HbA1c) of 5.3%, and low-density lipoprotein (LDL) 292 mg/dL. He was urgently referred to nephrology, but before he could be evaluated he began to develop edema in all four extremities, as well as facial edema, which prompted another ER visit. Initial workup included an albumin value of 0.8 g/dL, CTPA showed multiple bilateral lower lobe PE, and CT abdomen/pelvis revealed bilateral non-occlusive renal vein thrombi (Figure 1). He was placed on a heparin drip, evaluated by interventional radiology, and subsequently underwent bilateral renal vein thrombectomy. The urine protein/creatinine ratio indicated 11 g proteinuria, establishing the diagnosis of nephrotic syndrome. In the setting of bilateral renal vein thrombosis, membranous nephropathy was the highly suspected etiology. The patient was placed on high-dose steroids, an angiotensin-converting enzyme (ACE) inhibitor, a statin, and diuretics. A biopsy of the kidney was not done because the patient was seropositive for anti-phospholipase A2 receptor (PLA2R) antibody, conforming diagnosis of membranous nephropathy.

## Discussion

MN can be classified as either primary or secondary disease. It is associated with an increased risk of VTE compared to the other causes of nephrotic syndrome [2-9]. Secondary MN etiologies include hepatitis B and C, drug reactions, lupus, and other autoimmune conditions. PLA2R antibodies are present in 50-80% of cases, such as in the patient discussed above, and seropositivity is diagnostic for the disease [1]. The diagnosis of MN and the contributing factors are important in providing timely diagnosis, medical management, and preventing progressions to end end-stage renal disease [1,5]. Approximately 30% of patients with MN will progress to ESRD [6]. With the risk of ESRD and VTE, it is important to identify MN, including in the setting of limited laboratory abnormalities such as isolated hypoalbuminemia. Major clinical findings include proteinuria, periorbital edema, hyperlipidemia, hypoalbuminemia, and hypercoagulability [5]. 

VTE and arterial thromboembolic (ATE) events can be the initial presenting symptoms in patients with undiagnosed nephrotic syndrome, more commonly in MN [5,7]. The patient in this case report presented multiple times over a year with recurring PEs despite the use of anticoagulation and surgical intervention, with outpatient follow-up, laboratory abnormalities of hypoalbuminemia, hyperlipidemia, and proteinuria presented at different points in time. Severe hypoalbuminemia and facial edema were the presenting signs and symptoms leading to the hospital admission when MN was diagnosed.

VTE risk in patients with nephrotic syndrome varies depending on the underlying etiology, with the highest risk in MN, intermediate in focal segmental glomerulonephritis (FSGS), and lowest in IgA nephropathy (IgAN). In a study involving 1313 patients (395 with MN, 370 with FSGS, 548 with IgAN), the frequency of VTE was shown to be highest in MN (Table 1). The risk of VTE was directly proportional to the severity of proteinuria and inversely related to the albumin level. A 3.9-fold increased risk was found with serum albumin of <2.8 g/dL, and a 5.8-fold increased risk with serum albumin <2.2 g/dL [4]. In a study involving 898 subjects with MN, 7.2% had a thromboembolic event. Each 1.0 g/dL reduction in serum albumin was associated with a 2.13-fold increased risk of VTE. An albumin level <2.8 g/dL was the threshold below which the risk for a VTE was greatest [3].

**Table 1:** The Occurrence of Venous Thromboembolic Events in Patients with Different Subtypes of Nephrotic Syndrome FSGS: Focal segmental glomerulosclerosis; IGAN: IgA nephropathy; MN: membranous nephropathy; VTE: venous thromboembolism; DVT: deep vein thrombosis; PE: pulmonary embolism; RVT: Renal vein thrombosis Results are derived from a study by Barbour et al. [6]

	Total	FSGS	IGAN	MN
VTE	44	11	2	31
DVT	10	4	1	5
PE	20	8	1	11
RVT	19	2	0	17
Albumin	3.3	3.3	3.8	2.7

Currently, the Kidney Disease: Improving Global Outcomes (KDIGO) guidelines provide support for prophylactic anticoagulation in patients with nephrotic syndrome depending on TE and bleeding risks; however, further studies should be considered to provide an evidence-based approach in these patients at greater risk for TE and other negative outcomes [8]. In a study of 79 patients with biopsy-proven primary MN, 44 were placed on prophylactic anticoagulation. The 44 placed on prophylaxis had more severe hypoalbuminemia. There were five TE events in the non-anticoagulated group, but an increase in bleeding episodes in patients receiving anticoagulation. Due to the hypercoagulable state that can be found in patients with MN, there is a consideration for prophylactic anticoagulation in patients with severe hypoalbuminemia [9]. In this particular case, the patient was placed on an oral anticoagulation regimen in response to known TE, but still presented with multiple embolic lesions. Though KDIGO has made no official recommendation on the initiation and duration of anticoagulation, studies have shown to maintain anticoagulation as long as the patient is nephrotic, clinically at a higher risk for VTE due to other comorbidities, and do not have a contraindication such as a history of a hemorrhagic event or increased risk of bleeding [8]. Further studies need to be conducted to assess the benefit of preventing TE against the bleeding risks associated with anticoagulation [4,7-9]. 

Clinically significant TE should be assessed and the underlying etiology be understood in order to treat patients and prevent future episodes. Not all patients demonstrate clinical symptoms until a complete occlusion of vasculature is present [3,6]. The renal vein thrombi found in the patient above were non-occlusive, but the presence on imaging prompted further investigation due to the multiple PE and worsening symptoms associated with nephrotic syndrome. Whether routine screening for renal vein thrombi via CTA, MR angiography, or spiral CT is beneficial is still being studied further. If the imaging study is positive for renal vein thrombi there is an increased risk for PE [8].

## Conclusions

VTE are a common occurrence in the hospitalized setting and it is important to recognize nephrotic syndrome as one of the driving factors. The association of nephrotic syndrome and increased risk of venous thromboembolism is evident. The incidence of VTE are greatest at lower albumin levels, 2.0-2.5. Glomerular podocyte foot process effacement leading to hypoalbuminemia and decreased anticoagulation factors drives the liver to synthesize more procoagulable factors, leading to an adverse cycle of VTE events in patients. This requires awareness among medical professionals to be cognizant of the significant loss of protein and that hypoalbuminemia can be a useful signal of increased thromboembolic complications that require medical intervention. This case report presents a patient with multiple episodes of VTE, which could benefit from prophylactic use of anticoagulants to prevent VTE.
